# Development of a Confocal Optical System Design for Molecular Imaging Applications of Biochip

**DOI:** 10.1155/2007/79710

**Published:** 2007-08-30

**Authors:** Guoliang Huang, Shukuan Xu, Jiang Zhu, Cheng Deng, Zhonghua Dong, Yang Yang, Xiaoyong Yang, Xianhua Wang, Guofan Jin

**Affiliations:** ^1^Medical Systems Biology Research Center, School of Medicine, Tsinghua University, Beijing 100084, China; ^2^National Engineering Research Center for Beijing Biochip Technology, Beijing 102206, China; ^3^Department of Precision Instruments and Mechanology, School of Mechanical Engineering, Tsinghua University, Beijing 100084, China

## Abstract

A novel confocal optical system design and a dual laser confocal scanner have been developed to meet the requirements of highly sensitive detection of biomolecules on microarray chips, which is characterized by a long working distance ($w_{d}>3.0\;$mm), high numerical aperture (NA=0.72), and only 3 materials and 7 lenses used. This confocal optical system has a high scanning resolution, an excellent contrast and signal-to-noise ratio, and an efficiency of collected fluorescence of more than 2-fold better than that of other commercial confocal biochip scanners. The scanner is as equally good for the molecular imaging detection of enclosed biochips as for the detection of biological samples on a slide surface covered with a cover-slip glass. Some applications of gene and protein imagings using the dual laser confocal scanner are described.

## 1. INTRODUCTION

Systems biology is a discipline that examines the organizational relationships between biological structures in an organism, and thus it covers a very broad scope of biology from the macroscopic to the microscopic biology of organisms, ranging from mankind and other animals, plants, and microorganisms to their organs, tissues, cells, and subcellular organelles and structures through to molecular structures and the interactions between different molecular structures and systems. This broad scope of systems biology demands many different types of instruments for different aspects of imaging and signal detection. Instruments and devices such as cameras and X-ray imaging systems are used for whole organism imaging in man and larger animals [1, 2], IVIS imaging system has been developed for small animal imaging in vivo [3], ultrasonic devices are applied for the organ and tissue imaging [4], the ultraview living cell imager has been developed for cells and subcellular imaging [5], and various types of biochip have been designed for the analysis of the tissue, cells, and molecular structures [6]. Except for ultrasonic devices, all of the above detection-analysis systems require excellent optical design for the best performance of their particular objectives. One important type of optical system useful for the analysis of subcellular and molecular structures is the confocal chip scanner with a low background noise.

Advanced biochip analysis platforms [7, 8] analyze the content of a particular microarray slide chip, including gene chips, protein chips, cell chips, tissue chips, and others. Many researchers work with small molecule biochips, used for the detection of DNA variations by DNA hybridization, of proteins by the immunoreaction of proteins, and other more specialist analyses such as for DNA sequencing, ligase chain reaction (LCR), and others. Some important biochip detection systems [9, 10] have been developed, such as the fluorescence microscope imaging system and the laser confocal scanner. In all these detection systems, optical design has played a keyrole in obtaining high-clarity images of microscopic objects, and the optical objective is crucial to the detection sensitivity, the resolving power, and the working distance of the detection system. Usually, the bigger the numerical aperture of the objective, the higher its resolving power, and the higher the power of collecting the fluorescence signals bound on the tested object, however to achieve this structure, the working distance of the objective must also be shorter. For example, when the conventional microscope objective has an NA > 0.6, then its working distance *w_d_* is usually smaller than 1 mm. For example, the Zeiss plan objective 440050, magnification 40×, NA=0.65, $w_{d}=0.6$ mm, and the Nikon plan objective model 40×, NA=0.65, and $w_{d}=0.57$ mm.

In this paper, a novel confocal optical system design and a dual laser confocal scanner are described in which the materials and lenses employed are as small as practicable; the optical design and the potential pitfalls of an objective with a long working distance and a high numerical aperture have been considered and resolved for application to biochips. The collecting fluorescence has very high efficiency, and generally the instrument has excellent resolving power and an excellent signal-to-noise ratio.

## 2. CONFOCAL OPTICAL SYSTEM DESIGN AND ANALYSIS

### 2.1. A newly developed confocal optical system design

The design of a confocal optical system with high-performance detection of biological sample slides and biochips centers around the image quality, including the consideration of the resolution, zoom, aberration, and optical transfer functions, each of which can be optimized by using rays tracing calculations. In the process of optical design, all parameters of the optical system structure, including the surface curvature radius of each lens, the thickness of the lenses, the transmitted materials used, the separation distances, and the surface apertures can be varied to obtain an excellent optical specificity and image quality. The functional relationships between the specificity, image quality, and structural parameters of the system can be described as follows:

(1) $$\begin{array}{c} \xi_{1}\left(\eta_{1},\ldots ,\eta_{i}\right)=\psi_{1}\\ \vdots \\ \xi_{k}\left(\eta_{1},\ldots ,\eta_{i}\right)=\psi_{k}, \end{array}$$

where *i* and *k* are two natural numbers, $\psi_{1}\cdots \psi_{k}$ are various aberrations of the optical specificity and the image quality, $\xi_{1}\cdots \xi_{k}$ are functions of the specificity, the image quality, and the structural parameters of system, $\eta_{1}\cdots \eta_{i}$ correspond to all the structural parameters of system. Based on the polynomial expansion and the minimum binary iterative method, when all structural parameters $\eta_{1}\cdots \eta_{i}$ are modified repeatedly, an approximate minimum aberration will be obtained in (1).

**Figure 1:**
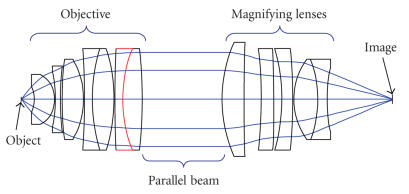
The main optical structure design of the confocal system.

Applying the above optical design method, an optimum confocal optical system structure for the detection of biochips could be defined, as shown in Figure 1. Here, the optical imaging system is composed of sets of objective and magnifying lenses. The objective system is a combination of seven lenses including two doublets and uses only three glass materials, ZK7, ZK11, and ZF2. The objective system has a high numerical aperture of 0.72 for collecting the signal from the object, a focal length of 13.06 mm, and a front focal length of 3.22 mm to provide a working distance of approximately 3.0 mm. There is a parallel ray path between the objective and the magnifying lenses, to which it is convenient to also add the filters and the dichroic mirror for the incident laser when building the confocal scanning system. The magnifying lenses system consists of five lenses. This optical system has a zoom of 3.

**Figure 2:**
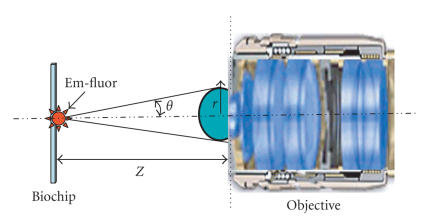
The configuration of the objective collecting fluorescence.

The optical structure parameters of confocal scanning system were optimized in Figure 1, where there are an objective and a magnifying lenses, the objective consists of seven lenses, the magnifying lenses consists of five lenses, there is a parallel beam between the objective and magnifying lenses. The optical structure parameters of confocal scanning system are listed in Table 1. A configuration of the objective collecting fluorescence is shown in Figure 2, where the distance from the focal plane to the front surface of first lens of objective is Z, *r* is the radius of effective aperture of the objective, θ is the half of aperture angle. The emission fluorescence (Em-fluor) of molecule bound on biochips is ideally a spherical wave, and the fluorescence bound on the biochip collected by the objective as shown in Figure 2 is described approximately by the formula

(2) $$I\left(Z\right)\approx \frac{K_{0}^{2}}{Z^{2}}\times \pi \times r^{2},$$

where $K_{0}$ is a constant, Z is the distance from the center of source in the focal plane to the first surface of objective, *r* is the radius of effective aperture of the objective.

When biochips are placed at the focal plane of the objective, and the objective has a numerical apture NA=n×Sinθ, where *n* is the refractive index, then the formula (2) can be simplified to formula

(3) $$I\left(Z\right)\approx \frac{1}{n^{2}}\times K_{0}^{2}\times \pi \times {\text{NA}}^{2}\times \left(1+{\text{NA}}^{2}+{\text{NA}}^{4}+\cdots \right).\;$$

Formula (3) indicates that the intensity of the collecting fluorescence of molecules on biochips has a direct ratio to the square of the higher power of the NA.

By developing a structure form using formula (3), we created a confocal optical system with a numerical aperture of 0.72, and an efficiency of collected fluorescence of more than 2-fold better than that of other commercial confocal biochip scanners [9, 10] whose objective has a smaller numerical aperture than 0.5.

### 2.2. Analysis of the new optical system design

**Figure 3:**
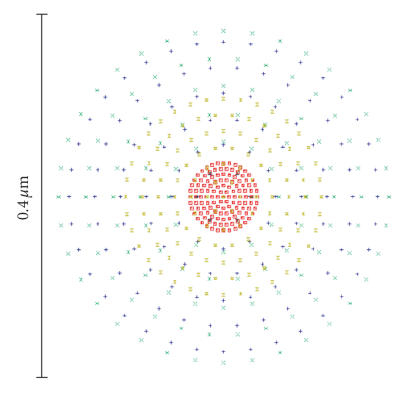
The spot diagram of the scanning probe.

In a confocal scanning system, the laser scanning spot probe determines the scanning resolution power. The smaller the laser scanning spot of optical system, the higher the scanning resolution power of the confocal system. The scanning spot diagrams are referenced to the real chief ray as shown in Figure 3. This option allows selection of two other reference points, the centroid and the middle. The centroid is defined by the distribution of the traced rays. The middle is defined so that the maximum ray errors are equal in both the *x*- and the *y*-directions. When a laser with a real beam diameter of about 1 mm is imaged by the objective shown above in Figure 1, then the spot diagram on the focal plane is as shown in Figure 3, where the scale is 0.4 μm, the spot diameter on the focal plane is smaller than 0.4 μm, which is an ideal scanning probe beam with a more high resolving power of <0.5 μm.

The optical speciality and the aberration of the imaging system of our novel optical structure can be analyzed using the optical design software ZEMAX-EE. For the spot diagram shown in Figure 3, the root mean square (RMS) radius on the focal plane is 0.105 μm, and the geometric radius on the focal plane is 0.183 μm, which corresponds to a confocal optical system with a scanning resolution power smaller than 0.4 μm.

**Figure 4:**
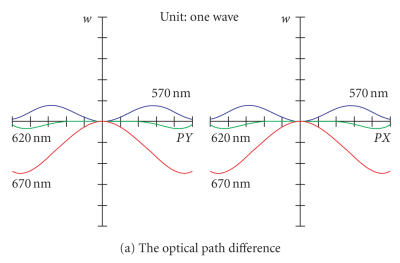

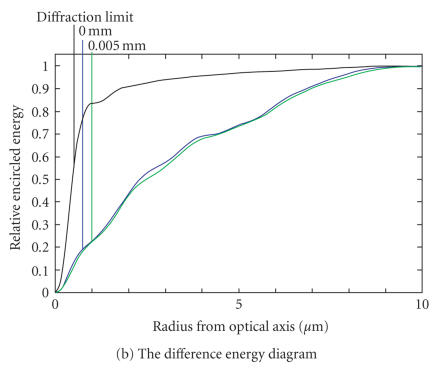
The optical path difference and the encircled energy diagram of the system.

The optical path difference (OPD) is a scalar quantity and it is identical to those for ray aberration fans at the tangential and sagittal directions PX and PY, respectively. The data plotted in Figure 4(a) is the optical path difference of the system in Figure 1, which is the difference between the optical path length of the ray and the optical path length of the chief ray. The horizontal scale of graph is the normalized entrance pupil coordinate. The vertical axis scale of graph is one wave, while the OPD maximum of system in Figure 1 is smaller than 5 waves. In Figure 4(a), there is a small optical path difference among 3 wavelengths 570 nm, 620 nm, and 670 nm, but the maximum optical path difference is smaller than 3 waves, which is lower than a normal visible light imaging system of 5 waves and can be used for confocal scanning system very well.

The encircled energy diagram is the percentage of total energy enclosed as a function of distance from either the chief ray or the centroid at the image of a point object, while the diffraction limit curve is for the aberration-free encircled energy computed on-axis. The encircled energy diagram of the system is shown in Figure 4(b), where the horizontal coordinate is the radius, and the vertical coordinate is the normalized fraction of the enclosed energy. The encircled energy diagram shows a diffused intensity spot in focal plane of system, the smaller the radius of encircled energy diagram is, the more the fluorescence energy is collected by the pinhole in the focal plane. In Figure 4(b), the radius of encircled energy diagram is smaller than 10 μm, where the efficiency of fluorescence collected is near to 100%, when a pinhole with a radius of 10 μm is set at the focal plane of the magnifying lenses, then the efficiency of collection of the fluorescence of an object is near to the diffraction limit.

**Figure 5:**
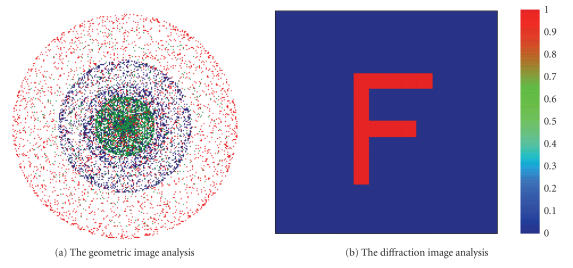
The geometric and diffraction image analyses of the system illustrated in Figure 1.

Geometric image analysis is used to model extended sources, to analyze useful resolution, to represent the appearance of imaged objects, and to provide intuition as to image rotation. The diffraction image analysis accounts for the finite pass band and other diffraction-related effects of real optical systems based upon Fourier Optics. The diffraction image analysis of the system is shown in Figure 5. The geometric image analysis of the optical system is shown in Figure 5(a), while the diffraction image analysis of the optical system in the object area illuminated by the scanning spot is shown in Figure 5(b), both of which have a high efficiency of 100%. In Figure 5(a), the geometric image analysis shows a nice roundness of model extended sources, there is good image rotation invariability of system. In Figure 5(b), the diffraction image analysis shows a nice uniformity and clear outline of the letter F as an object, there is good image quality of system to the object area illuminated.

### 2.3. The constitution of the confocal optical system

**Figure 6:**
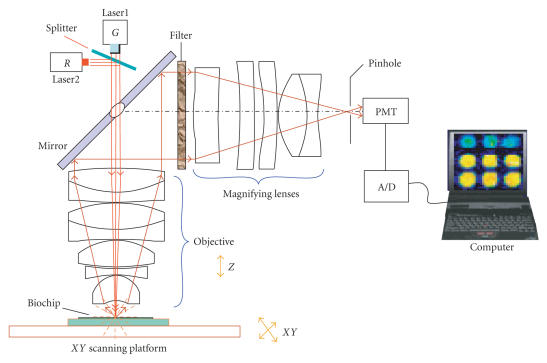
The newly developed confocal optical scanning system.

Applying the above optical design parameters, we constructed a new confocal optical scanning system which has been further developed into the advanced confocal scanner specially for biochip application, as illustrated in Figure 6, where the objective and magnifying lenses are designed in Figure 1, two filters for the dye Cy3 and the dye Cy5 are bought from Chroma Corporation, PMT (photomultiplier tube) is bought from Hamamatsu Corporation, laser 1 is a solid laser with wavelength 532 nm and power 25 mW, laser 2 is a semiconductor laser with wavelength 635 nm and power 25 mW, splitter is a dichroic mirror from Chroma Corporation, the mirror is machined into an elliptical mirror with a small hole 1mm in center, XY scanning platform is designed with 2 μm moving control precision. Pinhole is a small hole with diameter 20 μm, A/D electronic card is designed with precision 16 bit and frequency 1 MHz. Computer is chosen with PIII CPU or higher CPU speed, biochip is developed by CapitalBio Corporation. It is characterized by an objective with a large numeral aperture of NA=0.72, a long working distance of 3.0 mm, and a sensitivity of fluorescence detection of about 0.1 fluors/μm^2^. Compared to other similar commercial scanners, it has a higher-resolution power and an excellent signal-to-noise ratio. In Figure 6, when the laser beam irradiates the biochip on the XY scanning platform from the laser 1 with wavelength 532 nm, or from the laser 2 with wavelength 635 nm, the fluorescence of biological sample on the biochip is induced and collected by PMT. After A/D (analogue/digital) transfer is complete, the fluorescence signal from the biochip is loaded into the computer for digital image processing.

**Figure 7:**
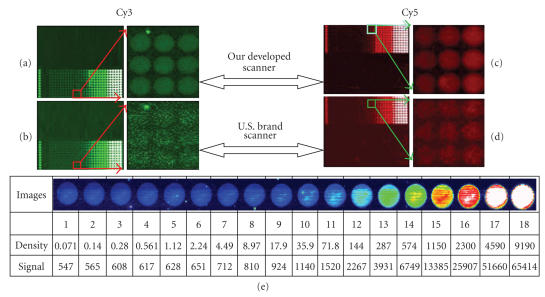
The analysis features of the newly developed confocal scanner.

When pairs of identical gradient signal biochips from Full Moon ( Full Moon BioSystems, Sunnyvale, Calif, USA) were labeled with either Cy3-tagged or Cy5-tagged probes, and dual color fluorescence was detected by using our newly developed confocal scanner and by another commercial confocal scanner (US popular brand S), the features of the scanning image analyses are shown in Figures 7(a)–7(d), where the right small spot array image is a local area magnifying view for left scanning images. Our newly developed confocal scanner shows a high scanning resolution, an excellent contrast, and a signal-to-noise ratio seen in Figures 7(a) and 7(c). The detection sensitivity of the new confocal scanner was further illustrated in Figure 7(e), where a Full Moon (Full Moon BioSystems, Sunnyvale, Calif, USA) normal molecular density biochip was used and the unit of density is the molecule number per the square micron, and the signal is the relative intensity in the range of 0 to 65 535. In Figure 7(e), a molecule with a density of 0.071 fluors/μm^2^ was detected with a signal of 547, where SNR (signal-to-noise ratio) is greater than 2, which indicates that the new confocal scanner is with a sensitivity of fluorescence about 0.1 fluors/μm^2^.

## 3. THE APPLICATION TO BIOCHIPS

### 3.1. The gene expression analysis

**Figure 8:**
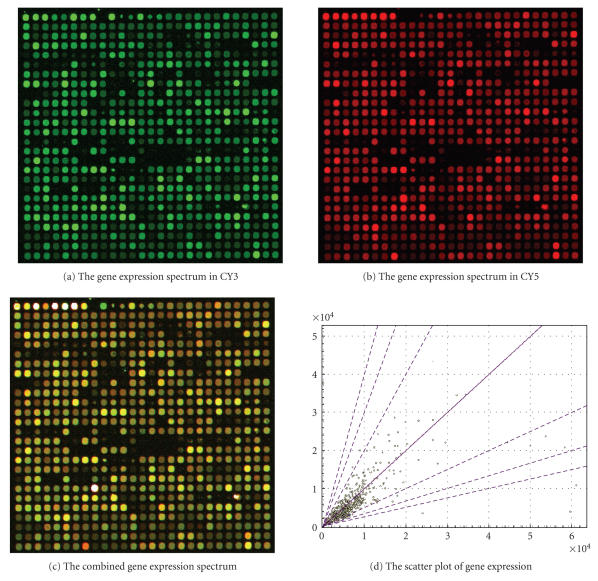
The gene expression spectra of a high-yield cotton compared to a common cotton variety.

The gene expression analysis is an important method to study the different gene functions of organisms, which is usually performed on high-density biochips with several tens of thousands of probes in a small 20 mm × 60 mm area, where dual fluorescent color labels with the dyes Cy3 and Cy5 can be used to produce a gene expression spectrum. In order to analyze the gene expression of biochips, it is important for the scanning system to possess high scanning resolution, an excellent contrast, and signal-to-noise ratio, over a wide range of signal intensities. The newly developed confocal optical scanning system can be readily used for the gene expression analysis of biochips. The gene expression spectrum for a high-yield variety of cotton as shown in Figure 8 was obtained by using the developed confocal scanner.

In Figure 8, the dual fluorescent color-labeled biochips were hybridized with 15 reference probes at the beginning of first row and with 687 gene reporter probes representing expressed genes from the two cottons. Messanger RNA from common cotton was labeled with Cy3, and mRNA from a high-yield cotton was labeled with Cy5. The dual fluorescent color-labeled biochips were scanned twice using the new confocal scanner, where one scan was illuminated by the green laser with a wavelength 532 nm to induce fluorescence of Cy3, and the second scan was illuminated by the red laser with a wavelength 635 nm to induce the fluorescence of Cy5. Figure 8(a) is the image of the gene expression spectrum of the common cotton labeled with Cy3, and Figure 8(b) is the image of the gene expression spectrum of the high-yield cotton labeled with Cy5. Figure 8(c) is the combined images of the gene expression spectrums from both the high-yield cotton and the common cotton, where there is an obvious color change if there is a difference between the level of gene expression of mRNAs labeled by Cy3 and by Cy5. Figure 8(d) is the scatter plot of gene expression to analyze the differences between the gene message labeled with Cy3 to message labeled with Cy5, where the identical level of expression of the same genes in the two plant varieties distributes along the line direction of 45 degrees with increasing relative intensity of signals. The larger the difference of gene expression between the two varieties, the farther the position of gene in the scatter plot is away from the 45-degree line. In Figure 8(d), except for 15-reference probes at row 1, there are 36 genes with a 2-fold signal difference between the common cotton and the high-yield cotton, distributed over a broad range of relative signal intensities, which shows that there are 36 important genes to improve the yield of cotton.

### 3.2. The detection of immunoreaction of proteins

**Figure 9:**
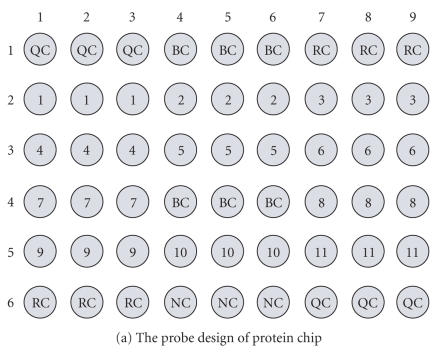

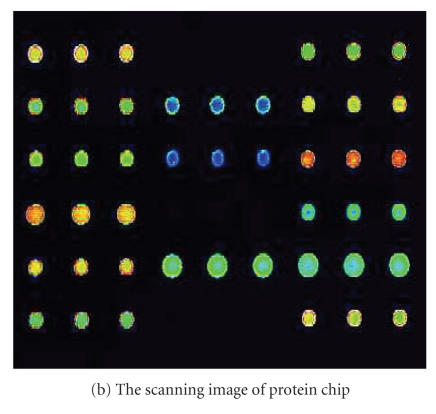
The application of protein chip for autoantibodies in patients serum.

The new confocal scanner is also useful for fluorescence detection of the immunoreactions of proteins on chips. We describe here a protein microarray chip for the parallel detection of autoantibodies in the serum of patients with autoimmune diseases, including systemic lupus erythmatosus (SLE), mixed connective tissue disease (MCTD), Sjögren's syndrome (SS), Sjögren's syndrome A (SSA), Sjögren's syndrome B(SSB), Smith (Sm), Ribonucleoprotein (RNP), Scleroderma (Scl), systemic sclerosis (SSc), dermotomyositis (DM), double-stranded DNA(dsDNA), Phosphate-Buffered Saline Tween-20(PBST), and polymyositis (PM). Purified autoantigens (SSA-52, SSA-60, SSB, Sm, RNP-68, Scl-70, Jo-1, dsDNA, centromere B, Ribosomal P0, and extracts of Hep-2 cells) are immobilized on the gel chip as shown in Figure 9(a), where QC—quality control, BC—blank control, RC—reaction control, NC—negative control, 1-Jo-1, 2-Sm, 3-Scl-70, 4-CENP-B, 5-dsDNA, 6-SSB, 7-SSA-52, 8-Extracts of Hep-2 cells, 9-SSA-60, 10-Ribosomal P0, 11-RNP-68. The protein microarray chip was incubated with 30 μL of a five diseases-mixed serum (diluted 1 : 100 with PBST) for 30 minutes at room temperature. After being rinsed and washed one time for 5 minutes with PBST, the chip was incubated with 30 μL of Cy3-labeled goat antihuman IgG antibody for 30 minutes at room temperature. After another rinse and 5-minutes PBST wash, the chip was briefly centrifuged to dry it. The mixed serum of SLE, SS, SSc, MCTD, DM, and PM positive sera patient was tested on the protein microarray chip, which was then scanned using our confocal scanner. The result of scanning image in Figure 9(b) clearly showed that this mixed sera contained anti-Jo-1 at row 2 from column 1 to column 3, anti-Sm at row 2 from column 4 to column 6, anti-Scl-70 at row 2 from column 7 to column 9, anticentromere B at row 3 from column 1 to column 3, anti-dsDNA at row 3 from column 4 to column 6, anti-SSB at row 3 from column 7 to column 9, anti-SSA-52 at row 4 from column 1 to column 3, antinuclear antibodies at row 4 from column 7 to column 9, anti-SSA-60 at row 5 from column 1 to column 3, anti-Ribosomal P0 at row 5 from column 4 to column 6, and anti-RNP-68 at row 5 from column 7 to column 9.

## 4. DISCUSSION AND CONCLUSION

This developed confocal scanner is good for some applications of biochips, such as DNA hybridization and immuno-reaction of proteins. The long working distance of the newly developed confocal scanner has a particular advantage for work with biochips enclosed with a hybridization gasket, or conventional microscope slides with thick covers, or even with an uncovered liquid surface. This feature is necessary for observation of real-time (RT) events on the chip surfaces, such as monitoring RT-fluorescence PCR. The high scanning resolution power available with a scanning beam of 0.4 μm and with a sensitivity of detected fluorescence of 0.1 fluors/μm^2^ are both important for obtaining an excellent images of small objects with quite clear definition, high contrast, and with a high signal-to-noise ratio. This improved clarity of the images could be seen when comparing scans of a Full Moon normal molecular density biochip by using our developed confocal scanner with that of using another commercial confocal scanner (see Figure 7).

When compared to other common microscope objective designs where the objective of a typical microscope has a numerical aperture of NA=0.65 and a working distance smaller than 1 mm (such as the Zeiss plan objective 440050, magnification 40×, NA=0.65, $w_{d}=0.6$, $w_{d}=0.6$ mm, and the Nikon plan objective model 40×, NA=0.65, $w_{d}=0.6$, $w_{d}=0.57$ mm), it is obvious that the optical design of the newly developed confocal scanning system is highly advanced, with an optimum combination of lenses, including a high numerical aperture of 0.72, a long working distance of 3.0 mm. The use of only 7 lenses and only 3 different kinds of optical glasses of ZK7, ZK11, and ZF2 is also beneficial. The limited number of glasses and lenses reduces the compound aberration in the use of large numbers of lenses and multiple glasses. In addition, these glass materials favor manufacture with small material error and small machining error.

Furthermore, the developed confocal scanner can be used for scanning cells and tissues slide, and because the high numerical aperture optical design has a very short focal depth 0. μ7m, it is also good for the tomography imaging of a cubic object.

## Figures and Tables

**Table 1:** The optimization data of confocal scanning system. Units are measured in mm.

Surface	Radius	Thickness	Glass	Semidiameter
1	−5.900	5.10	ZK11	2.80
2	−5.608	0.20	—	5.30
3	−23.000	1.20	ZF2	6.00
4	46.000	0.42	—	7.00
5	54.200	5.40	ZK7	8.50
6	−14.521	0.20	—	8.50
7	−106.500	2.00	ZF2	9.00
8	31.840	5.40	ZK7	10.75
9	−30.760	0.20	—	10.75
10	156.680	2.00	ZF2	10.75
11	24.720	4.90	ZK7	10.75
12	−79.250	100.00	—	10.75
13	25.650	5.51	ZK7	12.00
14	354.330	4.32	—	12.00
15	−73.450	3.15	ZF2	10.75
16	−183.900	2.00	—	10.75
17	−32.560	2.81	ZF2	10.75
18	−79.250	0.21	—	10.75
19	12.878	6.00	ZK11	8.50
20	−20.230	2.25	ZF2	8.50
21	26.300	—	—	—
